# Traumatic breast transposal to the abdominal wall: A case report

**DOI:** 10.3892/ol.2014.2303

**Published:** 2014-07-01

**Authors:** MEI YANG, ZHE XU, QIULIN LIAO, HONG WANG

**Affiliations:** 1 Breast Disease Center, Guangdong Women and Children Hospital of Guangzhou Medical University, Guangzhou, Guangdong 510010, P.R. China; 2 Breast Center, General Hospital of Guangzhou Military Command of the People’s Liberation Army, Guangzhou, Guangdong 510010, P.R. China; 3 Department of Ophthalmology, General Hospital of Guangzhou Military Command of the People’s Liberation Army, Guangzhou, Guangdong 510010, P.R. China; 4 Department of Pathology, General Hospital of Guangzhou Military Command of the People’s Liberation Army, Guangzhou, Guangdong 510010, P.R. China; 5 Department of Ultrasound, General Hospital of Guangzhou Military Command of the People’s Liberation Army, Guangzhou, Guangdong 510010, P.R. China

**Keywords:** abdominal wall mass, trauma, transposal breast

## Abstract

Although abdominal wall masses are commonly observed in clinical practice, traumatic breast transposal appearing as an abdominal wall mass is a rare event. The unique phenomenon of a post-traumatic breast growing healthily in the abdominal wall has never previously been reported. The current study presents the case of a 40-year-old female who developed an unusually transposed, but healthy mammary gland in the right upper abdominal wall following a severe pedestrian traffic accident. In that accident, the powerful impact of the car caused multiple right-sided rib fractures, lung injuries and a protruding mass on the right abdominal wall. This sudden onset protruding mass was indicated to be breast tissue by computed tomography imaging and ultrasound scanning. The transposed mammary gland was resected and a pathological examination confirmed that it consisted of normal breast tissue. In this case, the force of the car caused no significant damage or necrosis to the right breast, but instead was sufficient to shift the mammary gland to the abdomen, where it grew healthily 6 months in its new location. This case highlights the capability of the mammary gland to withstand a powerful impact and survive. Moreover, it advances our knowledge of how mammary tissues respond to severe blunt-force impacts.

## Introduction

Breast transposal/transplantation is rarely reported. Due to the increased use of mastectomy, much effort has been focused on breast reconstruction, including the use of adipose-derived stem cells (ASC) (1). Studies have used ASC for breast reconstruction (2) and augmentation (3). ASC assisted techniques can achieve satisfactory outcomes for breast augmentation (3,4), and Ribuffo *et al* (5) has reported the technique of lipofilling with ASCs following irradiation on the fill expander. However, to date, no encouraging results have been reported with regard to total breast reconstruction. The survival of foreign tissue or organs is difficult and therefore, tissue or organ transposal is rarely performed. In particular, transposal of an abdominal wall mass is an extremely rare event. Mammary gland transposal caused by blunt force has not been previously reported in the literature. Generally, when subjected to a powerful force, such as being hit by a car, and obtaining serious injuries to the chest wall, the breast may exhibit significant damage and necrosis. Even if the breast shifts to another position, without an adequate blood supply, the breast could not survive. Therefore, when normally confronted with an abdominal wall mass, a transposed breast would not be expected. Notably, in the present unique case, the force of a pedestrian traffic accident was sufficient to shift the mammary gland to the abdomen, where it grew healthily for 6 months in its new location.

## Case report

**Figure 1:**
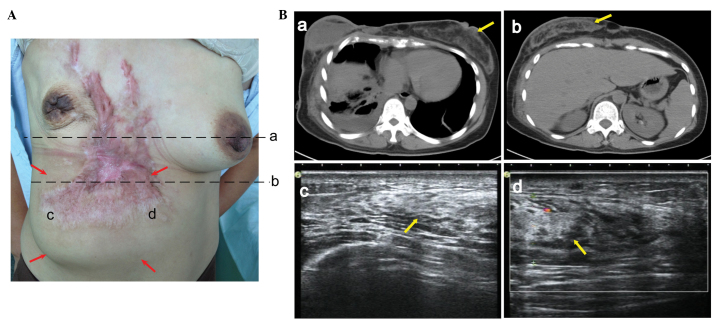
Initial examination. (A) Traumatic scar and right abdominal wall mass. Red arrows indicate the border of the mass. ‘a’ and ‘b’ dotted lines indicate the computed tomography (CT) scan level and ‘c’ and ‘d’ mark the ultrasound scan site illustrated in (B). (B) CT scan images for the (a) missing and (b transposed breast. (c and d) Representative ultrasound scan images of the mass. Yellow arrows indicate the mammary gland.

In July 2013, a 40-year-old female presented to the Breast Center, General Hospital of Guangzhou Military Command of the People’s Liberation Army (Guangzhou, China) with an unusual post-traumatic mass in the right abdominal wall was admitted by our department. The medical history was only notable for a car accident that had occurred 6 months earlier. In that car accident, the patient was provided with thoracic surgery to treat the rib fractures and lung injury. Following this, the patient was admitted to the Breast Center, General Hospital of Guangzhou Military Command of the People’s Liberation Army due to the sudden onset of a protruding mass on the right abdominal wall, which was indicated to be breast tissue by CT imaging. Upon examination, the right mammary gland was missing from its normal position, leaving a nipple areola complex on the right chest (Fig. 1A), and a mass ~11×15 cm in size was apparent in the right upper abdominal wall (Fig. 1A). This was further viewed by chest CT and ultrasound scans. The CT scan showed no right mammary gland on the right chest wall (Fig. 1Ba), but a mammary gland image of normal appearance was present in the right upper quadrant of the abdomen (Fig. 1Bb). Moreover, the ultrasound scan also indicated that the protruding mass on the abdominal wall was a mammary gland (Fig. 1Bc and d).

**Figure 2:**
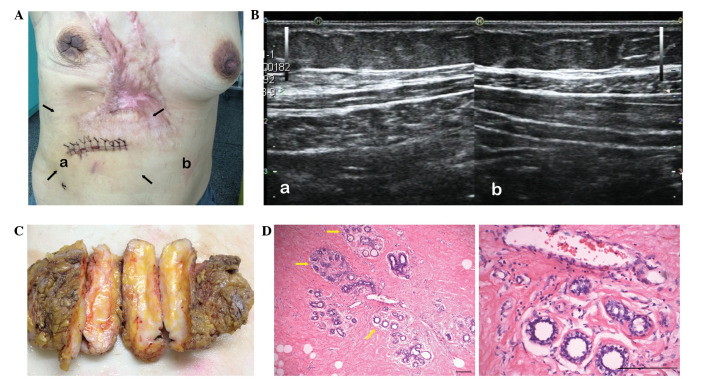
Following mass resection. (A) Abdominal appearance following complete resection of the abdominal wall mass. Black arrows indicate the border of the original mass. (B) Ultrasound imaging comparison of the (a) right and (b) left abdomen at approximately the same level following surgery. (C) Resected mass specimen. The specimen was transected, revealing the yellow and white interior. (D) Hematoxylin and eosin staining the showing microstructure of the resected specimen. Terminal duct lobular units (yellow arrows) can be clearly observed (magnification ×100, left; and ×400, right).

Reversion of the transposed breast to its original position was planned, however, the patient requested that it be removed. Therefore, a resection of the mass was performed resulting in complete removal of the transposed mammary gland (Fig. 2A and C). Following surgery, an ultrasound scan was performed again to ensure complete resection of the mass (Fig. 2B). The pathological diagnosis confirmed that the resected mass consisted of normal breast tissue (Fig. 2C and D) without notable necrosis. One month later, the patient returned for a follow-up examination, which showed that the abdominal wound had healed well. Consent was obtained from the patient for publication of this case study.

## Discussion

The most commonly reported tissue/organ transposals or transplantations include allogeneic hematopoietic stem cell transplantation to treat patients with hematopoietic cancer (6), autologous stem cell transplantation to treat liver volume expansion in patients with extensive hepatectomy (7), and allogeneic liver or kidney transplantations to treat end-stage liver (8,9) or kidney (10,11) disease. However, these procedures are performed manually, are complex and may cause complications, such as immune rejection. In addition, the tissue or organ is placed in the same position as its origin, providing a desirable environment for survival. Notably, the transplantation of ectopic pancreas tissue was reported by Endo *et al* (12) in Japan, which involved the removal of the tissue from its origin to a different environment. However, an adenocarcinoma arose from the ectopic pancreas tissue. Therefore, the survival of a normal ectopic organ/tissue is a rare event. To date, no whole breast transplantations have been reported, possibly due to the rare survival of ectopic normal organs/tissue.

Breast transposal is a rare condition, however, the present case study describes an original and unique case. In a traffic accident that occurred 6 months prior to admittance, the patient was hit by a car and thrown a large distance, resulting in multiple right rib fractures and lung injury. However, even following such as powerful impact, the right breast was not significantly damaged, and only the mammary gland shifted to the abdomen. This grew normally for ~6 months until the patient was admitted to the Breast Center, General Hospital of Guangzhou Military Command of the People’s Liberation Army. Even when considering the wide range of serious injuries that can occur to the chest wall, it is noteworthy that the patient developed a healthy transposed breast in the abdomen following such a severe trauma to the right chest. This case highlights the capability of the mammary gland to withstand a powerful impact and survive. This case suggests that breast transplantation may be possible in the future and also advances our knowledge of the manner in which mammary tissues respond to severe blunt-force impact by emigrating to a new location and surviving.
